# Impact of Aspiration Percutaneous Vertebroplasty in Reducing Bone Cement Leakage and Enhancing Distribution—An Ex Vivo Study in Goat Vertebrae

**DOI:** 10.3390/bioengineering10070795

**Published:** 2023-07-03

**Authors:** Hsin-Tzu Lu, Jia-Yi Lin, Yu-Chuan Tsuei, Yung-Fu Hsu, Chung-Yi Chen, Shih-Hao Cheng, William Chu, Chuan Li, Woei-Chyn Chu

**Affiliations:** 1 Institute of Biomedical Engineering, National Yang-Ming Chiao-Tung University, Taipei 11221, Taiwancli10@nycu.edu.tw (C.L.); 2 Department of Orthopedics, Cheng Hsin General Hospital, Taipei 11221, Taiwan

**Keywords:** osteoporotic vertebral compression fractures, percutaneous vertebroplasty, percutaneous kyphoplasty, aspiration percutaneous vertebroplasty, bone cement leakage, cement distribution

## Abstract

Osteoporosis-induced vertebral compression fracture (OVCF) occurs commonly in people over the age of 50, especially among menopausal women. Besides conservative therapy, minimally invasive percutaneous vertebroplasty (PVP) and kyphoplasty (PKP) have been widely used in clinical treatment and achieved good efficacy. However, the leakage of bone cement (CL) during vertebroplasty (PV) is a major risk that can cause (serious) complications such as compression of the spinal cord, pulmonary embolism, or even paraplegia. In this study, we introduced a new aspiration technique with standard PV procedures (APV) to ameliorate the risk of leakage with quantitative verifications of its effectiveness. APV intends to create a differential pressure to guide the direction of cement flow within the vertebrae. To test this technique, Nubian goats’ ex vivo vertebral bodies (VBs) were used to simulate the PV surgical process in humans. Results show that the proposed APV has a lower leakage rate of 13% compared to the 53% of conventional PV. Additionally, the APV approach achieves more uniform cement distribution via the 9-score method with a value of 7 ± 1.30 in contrast to 4 ± 1.78 by conventional PV.

## 1. Introduction

Osteoporosis is a condition that weakens bones and reduces bone mineral density and mass, leading to fragility and deformity, particularly in elderly and menopausal women [1,2]. According to the World Health Organization (WHO), the highest incidence of bone fractures due to osteoporosis is in the spine, i.e., the osteoporotic vertebral compression fractures (OVCF) [3]. In developed countries, the probability of suffering from osteoporotic fractures is around 30% to 40% in a lifetime, which is comparable to coronary heart disease [4,5]. In addition, untreated OVCF often brings about persistent pain, disability, being long-term bedridden, malnutrition, and even increased risk of mortality [6,7].

Typical clinical therapies of OVCF are conservative treatment and invasive surgeries. In general, pain relief is initially managed using conservative methods such as analgesics, bed rest, back brace, and anti-osteoporosis treatment for a duration of six weeks [8,9]. However, the failure rate of conservative treatments remains at 35–41% [10], and for failed treated patients, vertebral augmentation (VA) should be considered [11]. The two most common surgical interventions of VA are percutaneous vertebroplasty (PVP) and percutaneous kyphoplasty (PKP). Both procedures require the injection of polymethylmethacrylate (PMMA) cement into a compressed vertebral body (VB) to restore and maintain vertebral height [12,13]. A major difference between PKP and PVP is that PKP uses a balloon catheter to expand the collapsed vertebral body before the injection of bone cement, which is more manageable to restore and maintain the VB height during surgery [7]. The advantages of VA are that it is minimally invasive, relatively simple, and has lasting effectiveness. VA also quickly relieves pain and improves the quality of life of patients [14,15,16,17]. Nevertheless, a critical issue of VA is the leakage of bone cement (CL), with rates ranging from 56 % to 87.5% for PVP and 20% to 34% for PKP [14,16,18]. For the clinical study, a classification system was utilized to describe the patterns of bone cement leakage, which includes three types of leakage sites: (1) via the basivertebral vein (Type B), (2) via the segmental vein (Type S), and (3) through a cortical defect (Type C) [15]. According to a retrospective study, Type C occurs more commonly than Type B and Type S. [18]. Although many cases of extravasation may not cause symptoms, approximately 0.4% of extravasation can result in neurological complications, such as paraplegia or nerve root compression, when the bone cement leaks into nearby spinal canals and foramina [19]. Furthermore, the leakage of bone cement into blood vessels can potentially lead to fatal pulmonary embolism [15,16,17,18].

Given the above-mentioned risk, a promising surgical technique called aspiration percutaneous vertebroplasty (APV) was introduced for safer injection through PVP and PKP [20]. The technique utilizes an external sucking pump to create a pressure gradient between the bilateral trocars, which generates a force to guide the flow of bone cement within the VB, lowering the risk of CL. Unlike conventional PVP or PKP using a single trocar solely for bone cement injection, the procedure of APV introduces a second trocar to puncture at the contralateral side of the vertebra for the suction of bone cement by a pump. This technique creates a pressure gradient within the vertebra and thus helps to “draw” the flow of cement from the injection site to the sucking site. Results from previous studies show that the proportion of CL is greatly reduced, and the VB can be more uniformly filled. The uniformity of cement filling is important as several works of the literature showed that evenly distributed cement can relieve postoperative pain, prevent recompression of the treated VB, and avoid new compression fractures in adjacent VBs [21,22]. Another relevant issue is the filling pattern of bone cement. As cement continuously solidifies during the injection, a diffusive type (layer deposition) of bone cement during filling usually results in better long-term recovery than the dense type (bulk filling). Yu et al. reported that the dense-type filling may result in a 12.5-fold increase in the risk of re-collapsed augmented vertebrae following PVP treatment [23]. 

For animal models, the lumbar vertebrae of goats are considered anatomically and biomechanically similar to humans [24]. Many related studies have utilized goats as experimental subjects, including the development of a vertebral bone defect model [25], an experimental model for PV procedures [26], and the spinal mechanical stability after cement augmentation [27]. Accordingly, we choose goats as an ex vivo animal model for the evaluation of the efficacy of APV. Our objective is to compare APV with conventional PVP in the risk of bone cement leakage and improved uniformity of cement filling in VBs. To achieve this goal, several indices including the percentage of cement filling, leakage rate, and the uniformity of cement distribution in both vertical and horizontal directions within the VB are introduced to quantify the comparison.

## 2. Materials and Methods

### 2.1. Development of Ex Vivo Bone Models with Defects

#### 2.1.1. Preparation of Specimens

**Figure 1:**
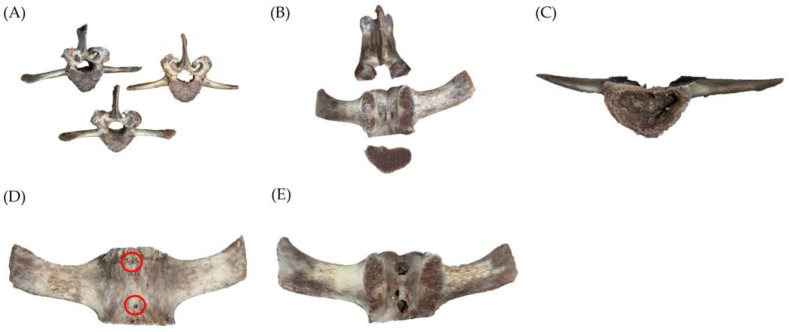
Photos illustrating the preparation processes of a goat vertebra. (**A**) These are some boiled and cleaned goat vertebrae that have been processed and used in this study. (**B**) From top to bottom: the vertebral arch, the vertebral body, and the posterior bone. Only the center vertebral body will be utilized in the experiment. (**C**) An intravertebral cavity is created by removing the trabecular bone. (**D**) Two leakage holes are created at the bottom of the vertebra (red circles) using a 1.5 mm drill. (**E**) The trocar insertion holes.

For this study, ex vivo goat vertebrae were utilized as a model for human vertebrae. Lumbar spine vertebrae from 14- to 20-month-old Anglo-Nubian goats were randomly assigned to control and experimental groups. The third to seventh lumbar vertebrae were selected for the experiment. The specimens were stored and frozen at −18 °C before experiments. To ensure that there were no biological infections during the experiment, the vertebrae were thawed and boiled to 100 °C. Then, soft tissues, e.g., muscles, cartilage, intervertebral discs, fats, etc., were completely removed from the bone, leaving only the VB for the bone cement injection experiment. Figure 1A shows a photo of the vertebra used in the experiment.

In order to simulate the cement injection in human vertebrae, the vertebral arch and the posterior bone were removed to allow for the insertion of trocars as illustrated in Figure 1B. This removal did not affect the experimental results. Additionally, the trabecular bone inside the vertebra was also removed to create a cavity for cement filling. The trabecular bone was intentionally removed to simulate the balloon-augmented cavity, which is typically created in a real surgical procedure using bone tamp inflation. It is important to note that in actual surgery, there is no need to remove the trabecular bone, as the cavity can be effectively created by inflating bone tamps (balloons). In our experiments, two electric drills were used, the Makita Driller and the Octopus Grinder, respectively. First, we used a 5 mm of the former to drill holes in the cancellous bone; then, we used a 4 mm of the latter to grind out an appropriate cavity. Afterwards, we used the Makita Driller to create 1.5 mm leakage holes and 5 mm puncture holes for trocar insertion, as illustrated in Figure 1C. The volume or cavity size was estimated by injection of water from a 3 mL syringe. 

#### 2.1.2. Vertebral Bone Defect Creation

Compression fractures in the vertebrae can cause cracks in the bone, which can lead to cement leakage during PV surgery. If the bone cement viscosity is too low or if the injection device applies pressure when the viscosity is high, cement may leak from the cracks, resulting in CL and postoperative complications. This study aims to investigate whether the APV process can reduce the risk of CL. To mimic the risk factor for Type C CL caused by cortical disruption, two leakage holes were drilled beneath the vertebra using an electric drilling machine with a 1.5 mm drill, as shown in Figure 1D [28]. 

Nevertheless, drilling holes above the vertebra was not performed as the bone in that area is more fragile, and the electric drilling machine used in the previous step could cause local damage to the bone. All holes below the lumbar vertebrae of the Nubian goat were used to examine the occurrence of CL during and after the cement injection process. Prior to the start of the experiment, the vertebra was placed on a dry surface and weighed on an electronic scale to obtain the “weight before injection.”

**Figure 2:**
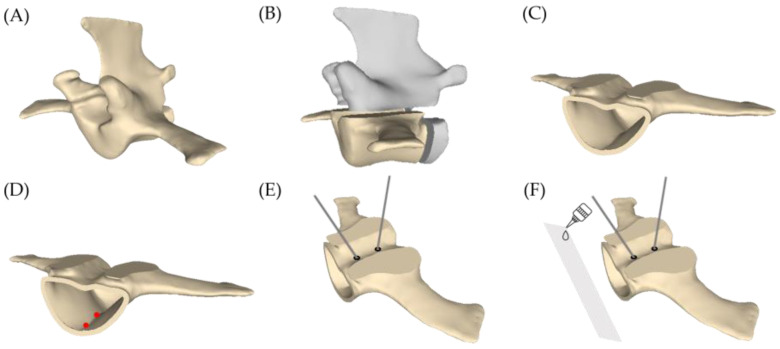
Schematic diagram of the vertebra preparation process. (**A**) A processed vertebra of a Nubian goat. (**B**) Removal of the upper and posterior bone (gray portion). (**C**) Elimination of trabecular bone to create the intravertebral cavity. (**D**) A 1.5 mm drill is used to create two holes at the bottom of the vertebra (red dots). (**E**) Two trocars are inserted and fixed by a photopolymerized resin. (**F**) A transparent plastic film is glued to the posterior side of the vertebra for visualizing the injected bone cement.

To simulate the clinical scenario of vertebroplasty, two trocars were inserted into both sides of the vertebral body using a 5 mm drill. The insertion holes were shown in Figure 1E. In this study, the trocars were fixed to the vertebrae using a photopolymerizing resin (PHC UV-5033, Taimoon, Taoyuan City, Taiwan). This step was performed before conducting experiments on the VBs of Nubian goats. The trocars were inserted into the VBs at a depth of half the width of the vertebrae. They were positioned at an angle of 60° relative to the vertical plane, specifically at the 2 o’clock position on the right or the 10 o’clock position on the left. A distance of approximately 2 cm between the two sheaths was used to achieve the pressure differential guiding effect. A transparent plastic film was then attached to the back of the vertebra using photopolymerizing resin, which enabled the flow of bone cement to be observed during the experiment and recorded with a video camera. The leakage holes were also checked to ensure that they were unobstructed, allowing for proper monitoring of leakage effects. Figure 2 is an illustrated schematic for handling VBs.

### 2.2. Equipment Setup and Pressure Pump Testing

Thirty vertebrae (L3–L7) will be included in this study and randomly assigned to either the control or experimental group. Both groups will be maintained in the same environmental conditions, with the laboratory temperature set at 17 °C to 20 °C, simulating the temperature of an operating room.

**Figure 3:**
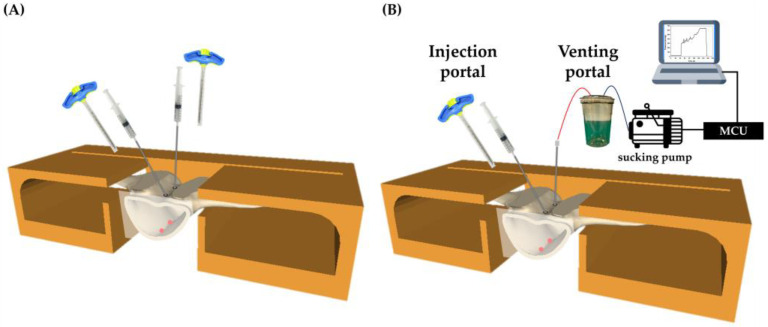
Description of the schematic setup for the PVP group and APV group. (**A**) PVP setup anchors the vertebra using a clamp, and cement is injected into the trocars on both sides. (**B**) APV set up. The cement is injected from the injection portal, while the other side (venting portal) sucks the cement to flow from the injection portal to the venting portal and, in the meantime, reads the pressure signals during the infusion process through the processor and displays the pressure change values on the computer.

For the PVP group, the vertebra was secured with a clamp, and both sides were used as injection portals, as illustrated in Figure 3A. During this process, cement was manually injected into the VB. In contrast, the APV group used one trocar as the injection portal, while the other trocar was connected to a filtering bottle and a sucking pump, serving as the venting portal. The sucking pump was connected to a microcontroller unit (MCU) that was programmed to monitor and display the real-time pressure within the vertebra, as shown in Figure 3B. To verify the initial experimental conditions, a water test will be conducted to ensure that the pressure can be maintained between 300 mmHg to 400 mmHg. The equipment will be set up, and if the water can be successfully suctioned into the bottle through the pressure differential and there is no water leakage from the two punctured holes underneath the vertebra, it indicates that the initial experimental condition is normal. If the above conditions are not met, it is necessary to re-examine the equipment or the experimental setup to identify problems. 

The setting time of high-viscosity acrylic bone cement (BondFix, XeliteMed, New Taipei City, Taiwan) is affected by temperature fluctuations during the initial mixing. Therefore, to ensure optimal injection efficacy, it is crucial to keep the bone cement powder and liquid components in an environment with a stable temperature between 17 °C and 20 °C for at least 30 min, as the setting time (cement hardening) of the used cement averaged around twelve and a half minutes, as per the cement instruction.

### 2.3. Bone Cement Preparation/Infusion/Cooling

Upon confirming the experimental environment and apparatus, the bone cement powder and liquid components will be mixed evenly for forty-five seconds in a sterile container. In our study, we utilized a front-opening cannula for cement injection. However, we want to emphasize that the APV technique can also be performed using a side-opening cannula, as long as the cannula’s outlet is directed towards the center of the VB. Both groups used intermittent injection to observe any CL. For the PVP group, cement injection will be stopped until a homogeneous dough is obtained at six and a half minutes post-injection. The total amount of cement injected through each trocar on both sides is 1.2 mL to 1.5 mL. If CL occurs, the injection will be immediately stopped. Otherwise, cement injection will continue until the estimated volume is reached. The estimated volume refers to the anticipated amount of bone cement intended to be injected into the vertebral body cavity. This estimation was made prior to the initiation of the injection procedure. The APV group will also monitor the pressure change during the injection process. The injection will be stopped when the pressure reaches the upper limit of 600 mmHg to 700 mmHg and remains there for 10 to 20 s. The infusion process is summarized in Table 1.

After the cement injection experiment, the trocars and the posterior transparent plate of the vertebra should be removed. Then, place the vertebra on an electronic scale to measure its “weight after the injection”. The difference in weight of the vertebra before and after the cement injection should be used to calculate the amount of cement that was injected into the vertebra. Once the cement has hardened, the vertebra should be cut in half to assess the filling percentage and distribution of the cement inside.

The measurements can be divided into the following three parts:

(1) Volume fraction percentage of VB:

(1) $$Cement\;Dispersion\;Volume\left(mL\right)=\frac{\mathrm{weight}\;\mathrm{after}\;\mathrm{in}\mathrm{ject}\mathrm{ion}-\mathrm{weight}\;\mathrm{before}\;\mathrm{injection}}{\mathrm{density}\;\mathrm{of}\;\mathrm{bone}\;\mathrm{cement}}\times 100\%$$

(2) $$Volume\;Fraction\;Percentage=\frac{\mathrm{cement}\;\mathrm{dispersion}\;\mathrm{volume}\;(\mathrm{mL})}{\mathrm{created}\;\mathrm{volume}\;\mathrm{of}\;\mathrm{cavity}\;(\mathrm{mL})}\times 100\%$$

**Figure 4:**
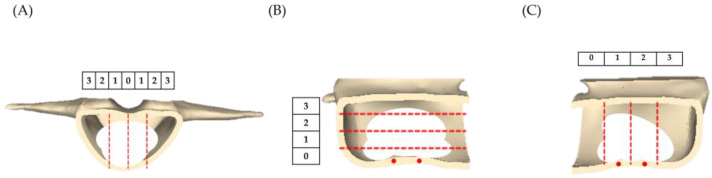
Diagram illustrating the criteria for the calculation of cement distribution inside the vertebra using the 9-score method. (**A**) Distribution along the vertical axis in the posterior view. (**B**) Distribution along the horizontal axis on the right side. (**C**) Distribution along the vertical axis on the left side. (White color: distribution of bone cement; red dots: 1.5 mm leakage holes; red dashed line: criteria for determining the distribution of bone cement.)

**Figure 5:**
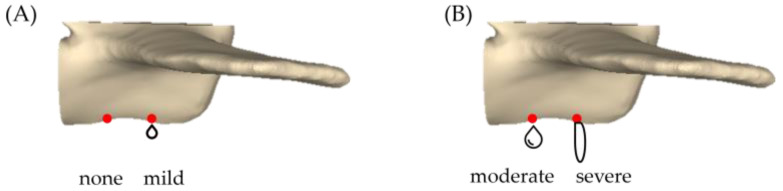
Degree of cement leakage. (**A**) None: no leakage observed from any hole at the bottom of the vertebrae; mild leakage: cement remained on the surface of the holes on the vertebrae but was not prominent. (**B**) Moderate leakage: cement slightly leaks out of the VB but with a volume less than 1 mL; severe leakage: cement leaked out of the vertebral body with a volume greater than 1 mL. (red dots: 1.5 mm leakage holes).

(2) Distribution of bone cement: The 9-score method is an objective scoring system used to assess the distribution of cement within the vertebra [29,30,31]. We calculate the scores of the vertical distribution in the posterior view, horizontal distribution on the right side, and vertical distribution on the left side of the vertebra. If the bone cement distribution was within 0% to 25% of the vertebra, it scores 0 points; within 25% to 50% scores 1 point; within 50% to 75% scores 2 points; and over 75% scores 3 points, as shown in Figure 4.

(3) Leakage rate of bone cement: Based on Churojana’s research, the degree of leakage has been adjusted [32]. The study measured the CL rate by examining artificially drilled holes created beneath the vertebra. The degree of CL was classified into four categories: no leakage, mild leakage, moderate leakage, and severe leakage, as depicted in Figure 5.

(3) $$Leakage\;Rate=\frac{\mathrm{number}\;\mathrm{of}\;\mathrm{cement}\;\mathrm{leakage}\;\mathrm{vertebra}\;\mathrm{per}\;\mathrm{group}}{\;\mathrm{total}\;\mathrm{number}\;\mathrm{of}\;\mathrm{vertebra}\;\mathrm{samples}\;\mathrm{per}\;\mathrm{group}}\times 100\%$$

### 2.4. Statistical Analysis

Statistical analysis was conducted using SPSS 17.0 for Windows (IBM Corporation, New York, NY, USA). The methods included descriptive statistics and a two-sample *t*-test. The *p*-value was set at 0.05.

## 3. Results

In this section, we describe the statistics and significant findings between the PVP and APV groups. The statistics of the data are summarized in Table 2. The experimental data suggest that the APV group had a higher filling percentage and more even distribution, as well as a lower leakage rate, when compared to the PVP group.

### 3.1. Volume Fraction Percentage

**Figure 6:**
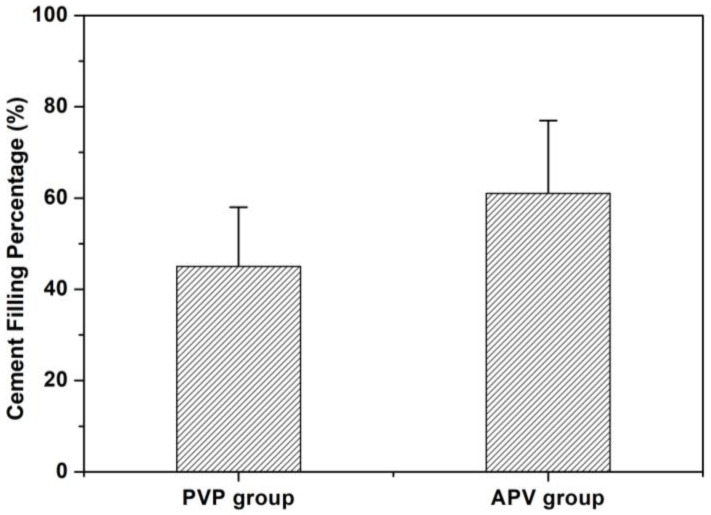
Volume fraction percentage of bone cement in study groups. The APV group was 61 ± 16%, while that of the PVP group was 45 ± 13% (mean ± standard deviation). There was no significant difference between the two groups (*p* > 0.05).

The volume of the artificially created cavity was 2.4 ± 0.42 mL and 2.7 ± 0.34 mL in the APV and PVP groups, respectively. Based on Equation (1), the cement dispersion volume in VB was calculated to be 1.45 ± 0.49 mL in APV and 1.20 ± 0.27 mL in PVP. Therefore, the volume fraction percentages according to Equation (2) were 61 ± 16% in the APV group and 45 ± 13% in the PVP group, as shown in Figure 6. However, there was no significant difference between the two groups (*p* > 0.05). 

### 3.2. Cement Distribution

**Figure 7:**
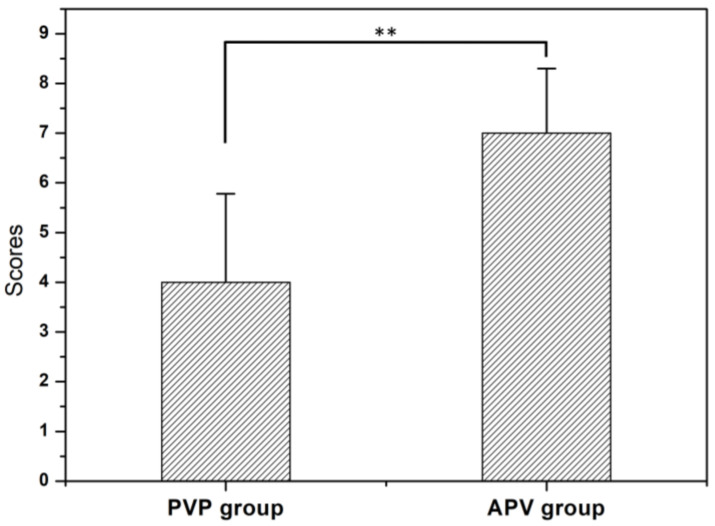
The scores of cement distribution in the vertebrae for each group. The average score was 7 ± 1.30 points for the APV group and 4 ± 1.78 points for the PVP group. The values are presented as mean ± standard deviation with a *p*-value greater than 0.01. ** *p*-value < 0.01, by two-sample *t*-test.

The 9-score method was used to assess the distribution of bone cement inside the vertebrae. The average score was 7 ± 1.30 points in the APV group and 4 ± 1.78 points in the PVP group, as shown in Figure 7. There were significant differences in the distribution of cement between the two groups (*p* < 0.01). From the cross-sectional view, the score of the APV group indicates that cement is more uniform in all three directions than the PVP group.

**Figure 8:**
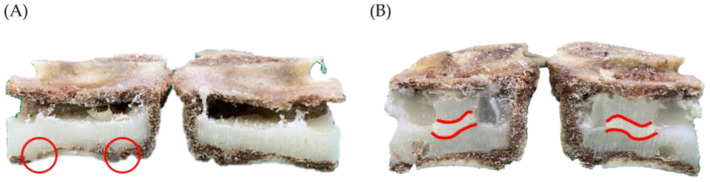
Demonstrate the cement distribution of the PVP group and the APV group. (**A**) In the PVP group, cement is deposited mostly in the bottom of the vertebral body due to gravity. In this case, only 0.88 mL of bone cement was injected before leakage occurred (leakage sites indicated by the red circles), leading to a volume fraction percentage of only 29%. (**B**) In the APV group, affected by the pressure gradient, cement appeared to be more evenly distributed within the vertebra. The two red lines in Figure 8B represent the layer-by-layer deposition of the injected bone cement. This deposition pattern can be observed by the color variation of the hardened cement in the coronal plane.

Based on the results, the PVP group is easily affected by gravity and was deposited in the lower half of the VB or forms adense type of two lumps of cement, resulting in a bottom-up filling circumstance. This can be observed in Figure 8A, where the cement began to accumulate in the bottom of the vertebra because of gravity. In contrast, due to the suction force, cement deposit for the APV group appeared more evenly distributed within the entire vertebra (Figure 8B).

### 3.3. Leakage Rate

**Figure 9:**
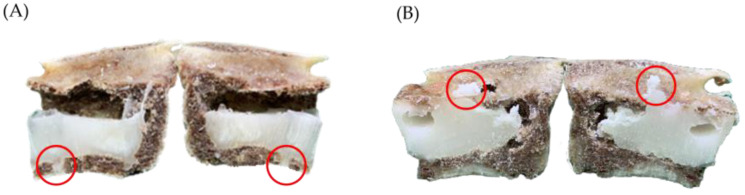
Cement leakage in the PVP group (**A**) and APV group. (**B**) The red circles denote the leakage sites. (**A**) The volume fraction percentage of this VB was only 41% in the PVP group. Cement can be observed to leak from the defect hole below and remain on the surface of the VB but was not significant. (**B**) For the APV group, the applied suction force gave a high percentage (82%) of cement deposit within the vertebra. However, there were still two cases of mild CL with cement extruding from the top holes of the vertebra. No leakage was observed from the lower leakage holes. This result shows “gravity” did not play a role in CL in the APV group, which can be accounted for by the inflicted pressure differential (leakage sites are indicated by the red circles). Note that the small cavities in (**B**) were caused by the removal of the inserted trocars.

In the PVP group, there were eight leakage cases, with a leakage rate of 53%. Among them, seven cases were mild leaks, where bone cement protruded vaguely at the defect holes, as seen in Figure 9A. This type is usually caused by gravity or too high injection pressure. The other leakage case was moderate due to bone cement extravasated visibly from the top hole of the vertebra. In contrast, in the APV group, there were only two leakage cases, and the leakage rate was 13%. There was no leakage observed from the lower defect holes. Instead, the CL occurred at the top hole, which was extruded by injecting excessive cement, as shown in Figure 9B. This result indicates that gravity had less influence on CL in the APV group, which could be attributed to the effect of aspiration vacuum.

## 4. Discussion

By injecting cement into the fractured vertebra for vertebral height restoration, PVP provides significant pain relief and improves the overall quality of life for OVCF patients [7,8,12,13,33]. The strength of the vertebral body and spinal stability can be substantially enhanced after this VA method [14,15,16,17]. However, a major drawback of this procedure is the risk of extra vertebral CL due to the uncontrollable pressure during injection, which has been reported to cause serious clinical complications such as spinal cord compression, radicular pain, systemic embolism, etc. [17,34].

Compared to the PVP group, in which no sucking pump was used, our results demonstrate that the application of a sucking pump significantly reduced the leakage rate from 53% to 13%, which is better than the previously reported leakage rates as high as 80% [14,16,18]. As the APV technique is less affected by gravity, it is less prone to CL than the PVP group. Several factors can contribute to CL, including the injection method, the volume of cement injected, the severity of bone defects, and the cement properties [35,36]. In clinical practice, apart from using high-viscosity bone cement, vertebrogram, or high-resolution fluoroscopy, the APV technique can be considered [37,38]. Occasionally, a sensor is used to detect the pressure changes inside the vertebra to detect the completion of the injection. This will prevent overfilling the vertebra, which can cause undesired CL. The advantage of using a sucking pump is that it can continuously create a pressure differential in the cavity, guiding the cement to fill the vertebra body and reducing injection resistance. This not only significantly reduces the risk of CL but also enhances the diffusion of cement, thereby improving the biomechanical support of the vertebra body.

In addition, the APV method has been used in many clinical applications for the improvement of PV efficacy. First of all, the introduction of suction can help aspire the content in the intravertebral cleft, which was beneficial for the diffusion of bone cement and lowered the incidence of subsequent fractures in the augmented level [39,40]. Second, pulsed jet lavage was performed by vacuum aspiration to remove the vertebral fat and bone marrow. This process before cement injection is effective to reduce CL, fat embolism, and enhance filling distribution [41,42,43]. Furthermore, suction can be used for tumor debulking through bilateral punctures. The vacuum-based suction is a safe, inexpensive, and reliable method for percutaneous intravertebral cavitation before VA [25].

Herein, to simulate the volume achieved by bilateral balloon inflation during PKP surgery, a cavity was created inside the VBs. Bilateral balloon inflation not only does not increase the risk during the operation but also has a better diffusion pattern and can restore the height of the VB [7]. In the APV group, the sucking pump generates a pressure differential that guides the flow of cement. An MCU connected to the sucking pump was programmed to detect pressure change and monitors the degree of cement injection in the VB. Our results indicate that APV can effectively assist the cement filling over 60% and avoid overfilling by real-time pressure monitoring. The higher filling percentage of the APV group is attributed to two factors. One is the amount of bone cement injection can be more than 1 mL of the cavity volume because less bone cement will be sucked into the external waste collection canister. The other is bone cement can be retained in VB and avoided extravasation, due to the vacuum aspiration force. According to the experiment protocol, when CL occurs, the injection will be immediately stopped. It is also worth pointing out that the aspiration by suction prior to the injection can reduce the pressure inside the VB cavity, which overall also improves the efficiency of cement filling.

Due to the influence of gravity, the cement in the PVP group tends to deposit at the bottom of the vertebra, resulting in an uneven distribution. In contrast, the APV can guide the cement by suction to deposit on the wall of the cavity. The newly injected cement would likely settle down on the previously deposited cement. This continuous process creates a layer-by-layer or diffusive-type filling. The outer layer solidified earlier than the inner layer, forming a protective shell to prevent potential CL. This approach also leads to a better and more symmetrical distribution of cement within the vertebra body, which has been demonstrated to improve postoperative clinical outcomes and lower the incidence of secondary fractures. It has been pointed out by several studies that the inhomogeneous distribution of cement can lead to an asymmetrical stiffness of the vertebra and local stress concentration, thereby increasing the risk of secondary fractures in adjacent vertebrae [44]. Therefore, a more evenly distributed cement within the VB is preferred as it can provide a better balanced mechanical state [45].

However, this study has some limitations. First, as an ex vivo experiment, it is difficult to simulate intraosseous pressure accurately. Second, clinical fractures are not the same as the manmade experimental leakage hole with a diameter of 1.5 mm, as a significant proportion of patients undergoing surgery experience more severe CL. Lastly, it is acknowledged that the process of preparing the vertebrae, which involves boiling and muscle removal, deviates from actual circumstances. However, it is important to emphasize that both the experimental and control groups in our study underwent the same preparation procedures. This deliberate approach ensures a consistent and fair comparison within the study, despite the variations from real-life conditions. Additional research, including animal and clinical trials, is required to confirm the safety and effectiveness of this technique.

## 5. Conclusions

In this study, a newly proposed APV technique, which utilizes a continuous sucking pump to maintain a pressure gradient within the vertebral body, can guide the flow of cement during the injection. Via ex vivo tests on goats’ vertebrae, this technique shows a lower risk of cement leakage and simultaneously achieves a more uniform distribution of cement in the vertebra cavity. Moreover, the diffusive (layered filling) and symmetrical pattern of cement distribution can provide better biomechanical support for the distressed VB. Nevertheless, it is important to note that further studies and clinical trials are necessary to validate the safety and efficacy of this technique in human patients.

## Figures and Tables

**Table 1:** The table lists the intermittent injection methods and end time of cement injection for the PVP and APV groups, respectively.

	PVP (Control Group)	APV (Experimental Group)
Injection method	About 1.2 mL to 1.5 mL of cement was injected into each side.	One side is injected with cement, while the other side is connected to the MCU to read the pressure values.
Termination timing ^+^	Continue the injection until the estimated volume is met.	Keep the injection until the pressure value reaches the 600~700 mmHg, and last for 10 to 20 s.

*^+^* Stop the injection immediately if cement leakage occurs.

**Table 2:** Comparison of data between the PVP and APV groups.

	PVP Group	APV Group	*p*-Value
Estimated cavity volume (mL)	2.70 ± 0.34	2.40 ± 0.42	0.22
Bone cement volume within the cavity (mL)	1.20 ± 0.27	1.45 ± 0.49	0.035 *
Weight of bone cement within the cavity (g)	1.80 ± 0.39	1.90 ± 0.65	0.015 *
Volume filling percentage of bone cement (%)	45 ± 13	61 ± 16	0.2
Average score using 9-score method	4 ± 1.78	7 ± 1.30	<0.01 **
Leakage rate (%)	53	13	-
Remark	Bone cement is filled from bottom to top	Bone cement is filled layer by layer	-

Values are presented as “mean ± standard deviation.” * *p*-value < 0.05, ** *p*-value < 0.01 was considered statistically significant.

## Data Availability

The data used to support the finding of this study are available from the corresponding author upon request.

## References

[1] Shen Y., Huang X., Wu J., Lin X., Zhou X., Zhu Z., Pan X., Xu J., Qiao J., Zhang T. (2022). The Global Burden of Osteoporosis, Low Bone Mass, and Its Related Fracture in 204 Countries and Territories, 1990-2019. Front. Endocrinol..

[2] Zhang S., Guo Q., Yang Y., Feng H., Zhao Y., Guo P., Li D., Du X., Song Q. (2023). Feasibility Study of 3D FACT and IVIM Sequences in the Evaluation of Female Osteoporosis. Bioengineering.

[3] Gao H., Huang J., Wei Q., He C. (2023). Advances in Animal Models for Studying Bone Fracture Healing. Bioengineering.

[4] Sözen T., Özışık L., Başaran N.Ç. (2017). An overview and management of osteoporosis. Eur. J. Rheumatol..

[5] Long Y., Yi W., Yang D. (2020). Advances in Vertebral Augmentation Systems for Osteoporotic Vertebral Compression Fractures. Pain Res. Manag..

[6] Prost S., Pesenti S., Fuentes S., Tropiano P., Blondel B. (2021). Treatment of osteoporotic vertebral fractures. Orthop. Traumatol. Surg. Res..

[7] Hsieh M.K., Chen W.J., Lee M.S., Lin S.Y., Liu M.Y., Lee D.M., Tai C.L. (2022). Biomechanical Evaluation of a Novel Expandable Vertebral Augmentation System Using Human Cadaveric Vertebrae. Appl. Sci..

[8] Liao J.C., Chen M.J.W., Lin T.Y., Chen W.P. (2021). Biomechanical comparison of vertebroplasty, kyphoplasty, vertebrae stent for osteoporotic vertebral compression fractures—A finite element analysis. Appl. Sci..

[9] Yu D., Liu Z., Wang H., Yao R., Li F., Yang Y., Sun F. (2022). Treatment of Elderly Patients with Acute Symptomatic OVCF: A Study of Comparison of Conservative Treatment and Percutaneous Kyphoplasty. Front. Surg..

[10] Zhang J.N., He X., Fan Y., Du J.P., Hao D.J. (2019). Risk factors for conservative treatment failure in acute osteoporotic vertebral compression fractures (OVCFs). Arch. Osteoporos..

[11] Castro A.P.G. (2021). Computational challenges in tissue engineering for the spine. Bioengineering.

[12] Schmidt-Rohlfing B., Reilmann H., Pfeifer R., Kobbe P., Pape H.C. (2011). Kyphoplastie und Vertebroplastie. Der Unfallchirurg.

[13] Fiani B., Newhouse A., Sarhadi K.J., Arshad M., Soula M., Cathel A. (2021). Special Considerations to Improve Clinical Outcomes in Patients with Osteoporosis Undergoing Spine Surgery. Int. J. Spine Surg..

[14] Wang B., Zhao C.P., Song L.X., Zhu L. (2018). Balloon kyphoplasty versus percutaneous vertebroplasty for osteoporotic vertebral compression fracture: A meta-analysis and systematic review. J. Orthop. Surg. Res..

[15] Semaan H., Obri T., Bazerbashi M., Paull D., Liu X., Sarrouj M., Elgafy H. (2018). Clinical outcome and subsequent sequelae of cement extravasation after percutaneous kyphoplasty and vertebroplasty: A comparative review. Acta Radiol..

[16] Zhan Y., Jiang J., Liao H., Tan H., Yang K. (2017). Risk factors for cement leakage after vertebroplasty or kyphoplasty: A meta-analysis of published evidence. World Neurosurg..

[17] Li Z., Yu K., Chang X., Cai S., Gao J., Wang Y. (2020). Cement leakage following percutaneous kyphoplasty in a patient after a posterior lumbar fusion: A case report. BMC Surg..

[18] Hsieh M.K., Kao F.C., Chiu P.Y., Chen L.H., Yu C.W., Niu C.C., Lai P.L., Tsai T.T. (2019). Risk factors of neurological deficit and pulmonary cement embolism after percutaneous vertebroplasty. J. Orthop. Surg. Res..

[19] Kong M., Xu X., Shen J., Liu Q., Wang G. (2019). Clinical characteristics and management of cardiac and/or pulmonary cement embolus after percutaneous vertebroplasty: A single center experience. Ann. Transl. Med..

[20] Chu W., Tsuei Y.C., Liao P.H., Lin J.H., Chou W.H., Chu W.C., Young S.T. (2013). Decompressed percutaneous vertebroplasty: A secured bone cement delivery procedure for vertebral augmentation in osteoporotic compression fractures. Injury.

[21] He S., Zhang Y., Lv N., Wang S., Wang Y., Wu S., He F., Chen A., Qian Z., Chen J. (2019). The effect of bone cement distribution on clinical efficacy after percutaneous kyphoplasty for osteoporotic vertebral compression fractures. Medicine.

[22] Sun H.B., Shan J.L., Tang H. (2021). Percutaneous vertebral augmentation for osteoporotic vertebral compression fractures will increase the number of subsequent fractures at adjacent vertebral levels: A systematic review and meta-analysis. Eur. Rev. Med. Pharmacol. Sci..

[23] Yu W., Xiao X., Zhang J., Li Z., Wang X., Tang F., Jiang X., Zhong Y. (2019). Cement distribution patterns in osteoporotic vertebral compression fractures with intravertebral cleft: Effect on therapeutic efficacy. World Neurosurg..

[24] Banstola A., Reynolds J.N. (2022). The sheep as a large animal model for the investigation and treatment of human disorders. Biology.

[25] Zhu X.S., Zhang Z.M., Mao H.Q., Geng D.C., Zou J., Wang G.L., Zhang Z.G., Wang J.H., Chen L., Yang H.L. (2011). A novel sheep vertebral bone defect model for injectable bioactive vertebral augmentation materials. J. Mater. Sci. Mater. Med..

[26] Benneker L.M., Gisep A., Krebs J., Boger A., Heini P.F., Boner V. (2012). Development of an in vivo experimental model for percutaneous vertebroplasty in sheep. Vet. Comp. Orthop. Traumatol..

[27] Tan E., Wang T., Pelletier M.H., Walsh W.R. (2016). Effects of cement augmentation on the mechanical stability of multilevel spine after vertebral compression fracture. J. Spine Surg..

[28] Pflugmacher R., Taylor R., Agarwal A., Melcher I., Disch A., Haas N.P., Klostermann C. (2008). Balloon kyphoplasty in the treatment of metastatic disease of the spine: A 2-year prospective evaluation. Eur. Spine J..

[29] Liu J., Tang J., Liu H., Gu Z., Zhang Y., Yu S. (2020). A novel and convenient method to evaluate bone cement distribution following percutaneous vertebral augmentation. Sci. Rep..

[30] Sun H.B., Jing X.S., Liu Y.Z., Qi M., Wang X.K., Hai Y. (2018). The optimal volume fraction in percutaneous vertebroplasty evaluated by pain relief, cement dispersion, and cement leakage: A prospective cohort study of 130 patients with painful osteoporotic vertebral compression fracture in the thoracolumbar vertebra. World Neurosurg..

[31] Liu J., Tang J., Zhang Y., Gu Z.C., Yu S.H. (2019). Percutaneous vertebral augmentation for osteoporotic vertebral compression fracture in the midthoracic vertebrae (T5-8): A retrospective study of 101 patients with 111 fractured segments. World Neurosurg..

[32] Churojana A., Songsaeng D., Khumtong R., Suwanbundit A., Saliou G. (2014). Is intervertebral cement leakage a risk factor for new adjacent vertebral collapse?. Interv. Neuroradiol..

[33] Liao P.H., Tsuei Y.C., Chu W. (2022). Application of Machine Learning in Developing Decision-Making Support Models for Decompressed Vertebroplasty. Healthcare.

[34] Chen J.K., Lee H.M., Shih J.T., Hung S.T. (2007). Combined extraforaminal and intradiscal cement leakage following percutaneous vertebroplasty. Spine.

[35] Wang W., Liu H., Wu Z., Teng Y., Huang Y., Liu T., Yang H. (2022). A Comparison of Percutaneous Kyphoplasty with High-Viscosity and Low-Viscosity Bone Cement for Treatment of Osteoporotic Vertebral Compression Fractures: A Retrospective Study. Geriatr. Orthop. Surg. Rehabil..

[36] Li M., Zhang T., Zhang R., Zhang H., Zhang D., Hu N., Wang Y. (2023). Systematic Retrospective Analysis of Risk Factors and Preventive Measures of Bone Cement Leakage in Percutaneous Kyphoplasty. World Neurosurg..

[37] Li K., Feng H., Luo D., Zhang W., Yang K., Ji C., Liu J., Xu H. (2020). Efficacy and safety of high-viscosity cement in percutaneous vertebroplasty for treatment of Osteoporotic vertebral compression fractures: A retrospective cohort study. Medicine.

[38] Chen W.C., Tsai S.H.L., Goyal A., Fu T.S., Lin T.Y., Bydon M. (2021). Comparison between vertebroplasty with high or low viscosity cement augmentation or kyphoplasty in cement leakage rate for patients with vertebral compression fracture: A systematic review and network meta-analysis. Eur. Spine J..

[39] Koike Y., Takizawa K., Ogawa Y., Fujikawa A., Yoshimatsu M., Nakajima Y. (2011). Percutaneous vertebroplasty for vertebral compression fractures with intravertebral cleft: Cement injection under vacuum aspiration. J. Vasc. Interv. Radiol..

[40] Li M., Zhang Y., Jin P., Jia P., Liu X.W., Tang H., Sun G. (2020). Percutaneous vertebral augmentation using drill rotation for osteoporotic vertebral compression fractures with intravertebral vacuum cleft. Skelet. Radiol..

[41] Albers C.E., Schott P.M., Ahmad S.S., Benneker L.M., Nieuwkamp N., Hoppe S. (2019). Vertebral body lavage reduces hemodynamic response to vertebral body augmentation with PMMA. Glob. Spine J..

[42] Piechowiak E.I., Isalberti M., Pileggi M., Distefano D., Hirsch J.A., Cianfoni A. (2019). Mechanical cavity creation with curettage and vacuum suction (Q-VAC) in lytic vertebral body lesions with posterior wall dehiscence and epidural mass before cement augmentation. Medicina.

[43] Yan J., Liu Q., Zheng Y., Liu Z., Liu X., Guo X., Liu P., Chen P., Yuan S., Tian Y. (2020). Effect of unilateral pulsed jet lavage prior to vertebroplasty on the intravertebral pressure and cement distribution. J. Orthop. Surg. Res..

[44] Li Q., Long X., Wang Y., Guan T., Fang X., Guo D., Lv J., Hu X., Jiang X., Cai L. (2021). Clinical observation of two bone cement distribution modes after percutaneous vertebroplasty for osteoporotic vertebral compression fractures. BMC Musculoskelet. Disord..

[45] Chen B., Li Y., Xie D., Yang X., Zheng Z. (2011). Comparison of unipedicular and bipedicular kyphoplasty on the stiffness and biomechanical balance of compression fractured vertebrae. Eur. Spine J..

